# Hidden diagnoses among patients with double seronegative myasthenia gravis

**DOI:** 10.3389/fneur.2026.1754393

**Published:** 2026-03-19

**Authors:** Vukan Ivanovic, Stojan Peric, Caitlin Briggs, Ana Marjanovic, Jovan Pesovic, Ivana Basta, Johan Jansson, Simrat Randhawa, Sonja Rajic, Sankalp Gokhale

**Affiliations:** 1 Neurology Clinic, University Clinical Center of Serbia, Belgrade, Serbia; 2 Faculty of Medicine, University of Belgrade, Belgrade, Serbia; 3 Dianthus Therapeutics, New York, NY, United States; 4 Faculty of Biology, University of Belgrade, Belgrade, Serbia; 5 Faculty of Medicine, University of Novi Sad, Novi Sad, Serbia

**Keywords:** congenital myasthenic syndrome, Lambert–Eaton myasthenic syndrome, myasthenia gravis, myotonic dystrophy type 2, seronegative

## Abstract

**Introduction:**

Double seronegative myasthenia gravis (dSnMG) is defined as myasthenia gravis (MG) without detectable antibodies to acetylcholine receptor (AChR) and muscle-specific kinase (MuSK). Absence of a disease-specific biomarker and clinical heterogeneity can significantly complicate diagnostic pathway. This study aimed to identify cases misdiagnosed as dSnMG.

**Methodology:**

The study included 33 patients [64% females, median age at onset 30 (22.5–40) years, median age at testing 46 (34–58) years] previously diagnosed with dSnMG. Disease severity was assessed using MG-ADL and QMG at testing, peak MGFA, intensive care unit (ICU) hospitalization and MG crisis history. Indirect immunofluorescence was performed to detect low-density lipoprotein receptor-related protein 4 (LRP4) antibodies. Whole exome sequencing (WES) was conducted, along with genetic testing for myotonic dystrophy type 1 and 2 (DM1 and DM2) and oculopharyngeal muscular dystrophy (OPMD).

**Results:**

Mean MG-ADL and QMG scores at testing were 1 (0–3) and 6 (3–9), respectively. More than half of the patients had ocular MG (52%). One patient experienced myasthenic crisis. One patient tested positive for LRP4 antibodies, and one was diagnosed with paraneoplastic Lambert–Eaton myasthenic syndrome. WES showed likely pathogenic variant c.517G > A in the *CHRNA1* gene associated with autosomal dominant slow channel congenital myasthenic syndrome and only one variant c.2368G > A in the *MUSK* gene. One patient displayed a DM2 premutation (32–35 CCTG repeats).

**Conclusion:**

This study highlights the importance of considering alternative diagnoses in patients with dSnMG and emphasizes the value of comprehensive testing. Early recognition of causative etiologies can significantly improve patient management and outcome and prevent unnecessary exposure to prolonged immunosuppression.

## Introduction

Myasthenia gravis (MG) is an acquired, antibody-mediated disorder of the neuromuscular junction, clinically characterized by fluctuating muscle weakness and fatigability (1). Based on autoantibodies targeting postsynaptic antigens, MG can be classified into acetylcholine receptor (AChR) (≈85%), muscle-specific kinase (MuSK) (≈6%), and low-density lipoprotein receptor-related protein 4 (LRP4) (≈2%) subtypes (2). When no circulating antibodies are detected using routine assays, but electrophysiological findings and/or response to acetylcholinesterase inhibitors and/or response to immunotherapy are consistent with MG, the condition is classified as seronegative MG.

Double seronegative MG (dSnMG) refers to patients without detectable antibodies against both AChR and MuSK. This subgroup accounts for approximately half of purely ocular MG cases and about 10% of generalized MG (gMG) (3, 4). However, the prevalence of dSnMG is likely overestimated when only conventional assays, such as enzyme-linked immunosorbent assay (ELISA), indirect immunofluorescence testing (IIFT), and radioimmunoprecipitation assay (RIA) are employed. The use of fixed and live cell-based assays (CBA), which allow detection of antibodies against clustered AChR, MuSK, and LRP4, significantly reduces the proportion of seronegative patients (5, 6). Patients who remain negative for AChR, MuSK, and LRP4 antibodies even after testing with CBA are classified as having triple seronegative MG (tSnMG) (7).

Seronegative cases represent the most diagnostically challenging MG subtype. Absence of a disease-specific biomarker may significantly delay diagnosis and complicate the diagnostic pathway, also leading to misdiagnosis (8). On the other hand, a false-positive MG diagnosis may result in unnecessary exposure to prolonged immunosuppression and delay appropriate treatment of the actual underlying condition. Therefore, it is essential to first consider and exclude various acquired and inherited disorders when diagnosing dSnMG.

We aimed to identify dSnMG misdiagnosed cases through detailed clinical, serological and genetic testing, including whole exome sequencing (WES), and testing for repeat expansion disorders that may mimic MG [oculopharyngeal muscular dystrophy (OPMD) and myotonic dystrophy type 1 and 2 (DM1 and DM2)].

## Patients and method

This study was approved by the Ethics Committee of the University Clinical Center of Serbia [#1141/2 from April 25, 2024]. Informed consents from all participants were obtained.

**Figure 1:**
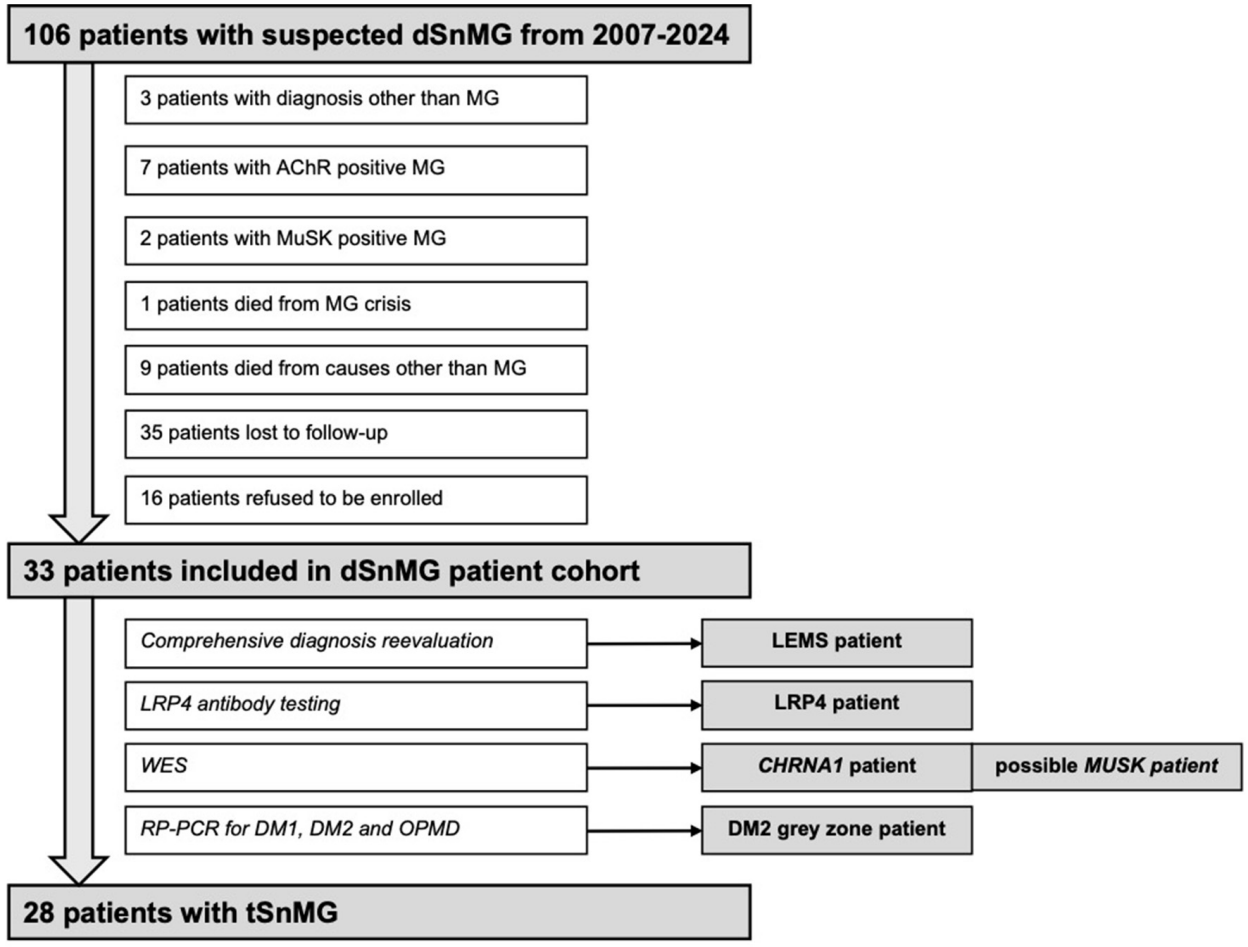
Flowchart showing dSnMG patients included in the study.

Data was collected through the electronic health-care information system of the University Clinical Center of Serbia. From 2007 to 2024, 106 patients initially diagnosed with dSnMG were found. Diagnosis other than MG was later confirmed in three cases (thyroid eye disease, congenital myasthenic syndrome (CMS) and ischemic stroke of medulla oblongata). Repeated antibody testing through the disease course detected anti-AChR antibodies in seven and anti-MuSK antibodies in two patients previously diagnosed with dSnMG, thus creating our initial cohort of 94 dSnMG patients. One of dSnMG patient died from myasthenic crisis, while nine more died from causes not related to MG (mainly cardiovascular and malignant diseases). Thirty-eight patients were lost from follow up, while 13 patients refused to enroll in the study. Finally, 33 dSnMG patients [64% females, median age at onset 30 (22.5–40) years, median age at testing 46 (34–58) years] were included. A flowchart of patient selection is provided in Figure 1.

All patients met the inclusion criteria for the study, which required: (1) characteristic clinical features (fluctuating muscle weakness and fatigability), (2) negative AChR and MuSK antibodies on RIA, and (3) at least two positive paraclinical criteria (A—pharmacological test (parenteral neostigmine or oral pyridostigmine) or B—characteristic electrophysiological findings (repetitive nerve stimulation test (RNS) or single-fiber electromyography (SFEMG) test that was applied only in those who were negative on RNS) or C—documented response to immunomodulatory therapy). We performed a detailed retrospective analysis of sociodemographic and clinical features, treatment regime and response, laboratory, electrophysiology, and other relevant data. Depending on the distribution of muscle weakness, we differentiated pure ocular and gMG. Disease severity was assessed using the Myasthenia Gravis Foundation of America (MGFA) pretreatment, at peak, and at enrollment, intensive care unit (ICU) hospitalizations and MG crisis, as well as Myasthenia Gravis Activities of Daily Living (MG-ADL) score, Quantitative Myasthenia Gravis (QMG) score, and Myasthenia Gravis Composite (MGC) score at enrollment (9–12).

In all patients, IIFT was performed to detect LRP4 antibodies and to establish a group of tSnMG patients (13). WES was performed to address potential genetic mimickers of dSnMG. WES was performed using Novaseq X (Illumina, San Diego, CA, USA) to capture and sequence almost all coding exons of ~20,000 known genes. Sequencing data analysis including the alignment to the Genome Reference Consortium Human Build (GRCh38) and Revised Cambridge Reference Sequence (rCRS) of the mitochondrial genome. Variant calling was conducted with open-source bioinformatics tools and software as previously described (14). It also incorporates Mutect2 v4.4.0 for calling lower level heteroplasmic variant in the mitochondrial genome, and ExpansionHunter v5.0.0 for calling repeat expansion variants. Common variants with allele frequency >5% in the gnomAD database12 (http://gnomad.broadinstitute.org) were filtered out except for the well-established disease-causing variants. Variant classification was done based on the American College of Medical Genetics and Genomics (ACMG) and the Association for Molecular Pathology (AMP) guideline (15). Patients' clinical phenotypes were transformed to corresponding standardized human phenotype ontology terms and accessed to measure symptom similarity with each of ~7,000 rare genetic diseases (16, 17). Finally, the variants were prioritized by the classification and symptom similarity score, and the medical geneticists manually evaluated the candidate variants and associated diseases. All variants were confirmed using bidirectional Sanger sequencing. In a patient with a heterozygous pathogenic variant in a gene associated with recessive CMS, we performed whole-genome sequencing (WGS) using the NovaSeq X platform (Illumina, San Diego, CA, USA). The WGS library was prepared using the TruSeq DNA PCR-Free kit. Repeat-primed polymerase chain reaction (RP-PCR) was applied for diagnosis of DM1, DM2 and OPMD addressing the limitation of WES in detecting repeat expansion disorders (18, 19).

Methods of descriptive statistics were applied: proportion, median and interquartile range.

## Results

**Figure 2:**
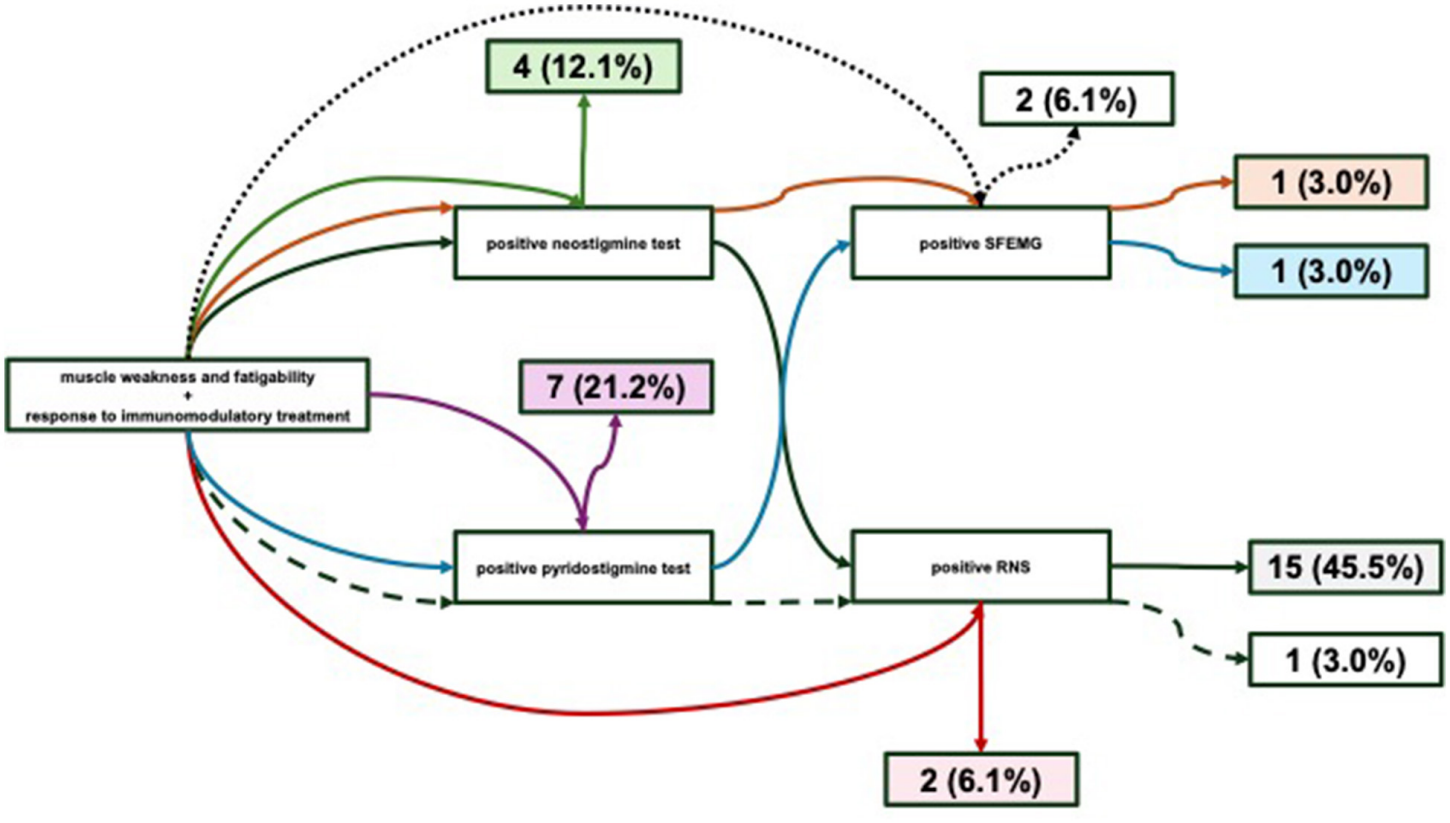
Criteria fulfilled to make diagnosis of dSnMG in our patients. All patients had features of muscle weakness and fatigability plus response to immunomodulatory treatment, with additional criteria: positive neostigmine test + positive RNS test in 15 (45.5%) patients (black arrows), positive neostigmine test + positive SFEMG in 1 (3.0%) patient (orange arrows), positive neostigmine test in 4 (12.1%) patients (green arrows), positive response to pyridostigmine + positive RNS test in 1 (3.0%) patient (dashed black arrows), positive RNS test in 2 (6.1%) patients (red arrows), positive response to pyridostigmine + positive SFEMG in 1 (3.0%) patient (blue arrows), positive response to pyridostigmine in 7 (21.2%) patients (purple arrows), positive SFEMG in 2 (6.1%) patients (dotted black arrows). RNS, repetitive nerve stimulation; SFEMG, single-fiber electromyography.

All 33 dSnMG patient from investigated cohort had typical clinical presentation of fluctuating muscle weakness and fatigability. Thorough analysis of their medical records showed response to immunomodulatory treatment in all of them. Pharmacological test was positive in 84.8% of cases, and electrophysiology in 66.7%. Detailed data on predefined dSnMG criteria are given in Figure 2.

Out of the 33 dSnMG patients enrolled, five (15.2%) were found to potentially have alternative diagnoses, with three (9.1%) having a confirmed different diagnosis (Table 1). One patient (3.3%) tested positive for LRP4 antibodies. In another patient (3.3%), detailed re-evaluation of medical records, showing an initial decremental response on RNS and a positive neostigmine test which led to the original dSnMG diagnosis, resulted in a revised diagnosis of Lambert–Eaton myasthenic syndrome (LEMS). This was supported by repeated electrophysiological testing demonstrating a 200% increment on high-frequency RNS, serum positivity for voltage-gated calcium channel (VGCC) antibodies, and the presence of small-cell lung cancer.

**Table 1:** Sociodemographic and clinical features of original dSnMG, misdiagnosed dSnMG patients and final tSnMG cohort.

**Patients/features**	**Original dSnMG cohort**	**Final tSnMG cohort**	**LRP4 patient**	**LEMS patient**	***CHRNA1* patient**	**Possible *MUSK* patient ^1^**	**DM2 gray zone patient ^2^**
*N*	33	28	1	1	1	1	1
Sex	36.4% males 63.6% females	35.7% males 64.3% females	Male	Female	Female	Female	Male
Age at onset (years)	30 (22.5–40)^*^	30 (22.5–39.5)^*^	52	56	12	39	28
Diagnostic delay (months)	6 (1.25–9)^*^	4 (1–9)^*^	12	12	6	6	5
Age at enrollment (years)	46 (34–58)^*^	45 (33.5–57.0)^*^	59	57	24	61	43
Positive electrophysiology test	66.7% positive	64.3% positive	Positive	Positive; later also 200% increment in RNS with high-frequency stimulation	Positive	Positive	Negative
Response to acetylcholinesterase inhibitors	84.8% positive	78.8% positive	Positive	positive	Positive	Positive	Positive
Thymus on CT or MRI	normal 48.5% residual 21.2% thymoma 3.0% not available 27.3%	normal 50.0% residual 21.4% thymoma 3.6% not available 25.0%	Normal	Enlarged MLN^1^	Normal	Not available	Residual
Thymus surgery	24.2%	24.0%	No	No	Yes	Yes	No
Generalized vs. ocular MG	ocular 51.5% generalized 48.5%	ocular 57.1% generalized 42.9%	Generalized	Generalized	Generalized	Generalized	Ocular
MGFA class before treatment	I 54.5% IIa 3.0% IIb 12.1% IIIa 6.1% IIIb 21.2% IVb 3.0%	I 60.7% IIa 3.6% IIb 14.3% IIIa 3.6% IIIb 17.9% IVb 0.0%	IIIb	IVb	IIIa	IIIb	I
MGFA class at peak	I 51.5% IIb 6.1% IIIa 9.1% IIIb 18.2% IVb 9.1% V 3.0%	I 59.3% IIb 7.4% IIIa 7.4% IIIb 14.8% IVb 7.4% V 3.7%	IIIb	IVb	IIIa	IIIb	I
MGFA class at enrollment	I 33.3% IIa.18.2% IIb 3.0% IIIb 9.1% Remission 36.4%	I 35.7% IIa 66.7% IIb 3.6% IIIb 7.1% Remission 39.3%	1	IIIb	IIa	IIb	Remission
MG crisis	3.0%	3.6%	No	No	No	No	No
ICU hospitalization	12.1%	10.7%	No	Yes	No	No	No
MG-ADL score at enrollment	1 (0–3)^*^	1 (0–3)^*^	5	10	0	2	0
QMG score at enrollment	6 (3–9)^*^	6 (3–7.75)^*^	13	18	8	6	1
MGC score at enrollment	1 (0–4)^*^	1 (0–4)^*^	7	19	0	4	0
Disease course			Initially presenting with severe bulbar symptoms. Treated with plasmapheresis. Bulbar symptoms completely responded to treatment. Ocular manifestations remained refractory	Severe disease course, with pronounced bulbar symptoms and limb weakness. She reported significant weight loss and had a history of tobacco use. Muscle reflexes were absent, but she did not have neither postexercise facilitation nor autonomic dysfunction. Serum antibodies against voltage-gated calcium channels (VGCC) were positive. Confirmed diagnosis of a small cell lung carcinoma	Normal early psychomotor development. At age of 12, fluctuating global muscle weakness in upper and lower limbs, and ptosis. Partial subjective improvement on immunotherapy, her symptoms remained largely refractory. Several months after thymectomy, her symptoms mostly resolved, except for the mild cervical and finger extensor weakness with transient ocular symptoms. Genetic diagnosis was made at the age of 23. She started on fluoxetine (20 mg three times daily), which significantly improved her neurological deficit	Patient developed bulbar and limb muscle weakness at age of 39. Bulbar symptoms resolved early after introduction of cholinesterase inhibitors and immunomodulatory therapy, while proximal lower limb muscle weakness persisted. Intermittent bulbar exacerbations. Her family history was unremarkable, although they have not been available for genetic testing	Pure ocular manifestations with a favorable response to therapy

^1^This patient has only one pathogenic variant in the *MUSK* gene that is associated with autosomal recessive congenital myasthenic syndrome. Thus, she has no confirmed genetic diagnosis. Still, we did not include this patient in a final triple seronegative group since it is possible that copy number variation that can be missed by genetic tests is present on another allele.

^2^Patient did not have more than 75 repeats in the *CNBP* gene which is needed to make diagnosis of DM2. However, since literature reports symptomatic patients with the number of repeats in a gray zone, this patient was not included in a final triple seronegative group.

^*^ Results are presented as median (interquartile range).

WES showed likely pathogenic variant c.517G > A in the *CHRNA1* gene associated with autosomal dominant slow channel CMS in one patient, and a pathogenic variant c.2368G > A in the *MUSK* gene in another. The patient carrying a *CHRNA1* gene mutation had normal early psychomotor development. At age of 12, she experienced fluctuating proximal than distal muscle weakness and ptosis with rare but occasionally severe exacerbations. Genetic diagnosis was made at the age of 23 when she was started on fluoxetine (20 mg three times daily) which significantly improved her neurological deficit. Her family history was negative for CMS, and all family members tested negative for the *CHRNA1* variant.

Patient carrying a heterozygous mutation in the *MUSK* gene developed bulbar and limb muscle weakness at age of 39. Despite immunotherapy, proximal lower limb muscle weakness persisted. Disease had a fluctuating course with intermittent bulbar exacerbation. Since *MUSK*-related CMS is inherited in an autosomal recessive manner, WGS was further performed, yet no second pathogenic variant was identified. Her family history was unremarkable, and they have not been available for genetic testing.

None of our patients tested positive for DM1 and OPMD, while one patient, presenting with pure ocular manifestations and a favorable response to therapy, was found to have a DM2 premutation (32–35 CCTG repeats). The remaining 28 patients constituted the group of patients with a confirmed diagnosis of tSnMG. A comparative overview of the sociodemographic and clinical characteristics of original dSnMG group, misdiagnosed dSnMG patients and confirmed tSnMG patients is presented in Table 1.

## Discussion

This study demonstrated that 15% of patients initially diagnosed with dSnMG were found to potentially have alternative diagnoses after comprehensive evaluation, with three cases (9%) being confirmed. This finding is consistent with previous reports describing frequent false-positive diagnoses of dSnMG, which may result in unnecessary prolonged immunosuppressive treatment and, in some cases, even surgical intervention, while the actual underlying condition remains untreated (20–22).

LRP4 antibodies were detected in only one patient (3.3%) in our dSnMG cohort, which is markedly lower than the prevalence rates of up to 30% reported in previous studies (23–25). Reduced frequency observed in our patients may be due to the well-documented lower occurrence of LRP4 antibodies among Caucasians and due to the limited sensitivity of IIFT compared to CBAs. LRP4 antibody-positive MG patients are typically considered to have milder disease course with a favorable outcome, often with isolated ocular symptoms (4, 23–26). Our patient initially presented with gMG and severe relapses that required rescue therapy, while refractory ocular symptoms persisted later. In line with our findings, Rivner and colleagues reported that LRP4 and/or agrin antibody positive patients more commonly present with generalized and severe disease compared to seronegative MG patients (27).

LEMS is a widely recognized MG mimic. Shared clinical phenotype, similar response on low frequency nerve stimulation and response to immunomodulatory and symptomatic treatment can significantly complicate the distinction between these two conditions, as was the case with our patient (28). Additional obstacle to make proper diagnosis in our patient was absence of autonomic impairment. Presence of constitutional symptoms, risk factors suggestive of malignancy and incomplete response to cholinesterase inhibitors, should raise suspicion of LEMS, even in the absence of hyporeflexia and pronounced dysautonomia (29). Consistent with our data, Kwon et al. found that 3% of patients suspected of having AChR-antibody negative MG were ultimately diagnosed with LEMS (30). Several other articles highlighted similar diagnostic challenges (31, 32). In such cases, the importance of timely diagnosis becomes particularly important, especially in paraneoplastic LEMS.

One out of our 33 (3%) dSnMG patients had genetically confirmed CMS, showing a slow-channel CHRNA1 mutation. *CHRNA1* mutations account for less than 3% of all reported CMS cases (22, 33). The differential diagnosis between dSnMG and CMS is particularly challenging in adult-onset CMS, where up to 50% of patients may initially be misdiagnosed with MG, compared to less than 10% in childhood-onset cases (22, 34). To the best of our knowledge, only a few studies have investigated misdiagnosed CMS patients within cohorts of dSnMG patients (20, 21, 35). Lorenzoni and colleagues analyzed the most common mutations in the *RAPSN, DOK7*, and *CHRNE* genes and identified a single positive case among 22 patients tested (4.5%), harboring compound heterozygous variants in CHRNE (20). In a study by Alseth and colleagues, the *RAPSN* and *DOK7* genes were screened for the N88K and c.1124_1127dupTGCC mutations. Among 74 dSnMG patients, one individual (1.4%) was homozygous for the N88K variant, and two additional patients were carriers of N88K. Sequencing of *DOK7* revealed no pathogenic variants (21). Such a targeted genetic approach would have failed to identify our slow-channel CMS patients. Thus, we highlight the advantages of WES, as demonstrated in the Australian cohort described by Garg et al. (35). They performed WES only in patients with initial diagnosis of adult-onset dSnMG and with clinically affected siblings, resulting in a 28% diagnostic yield (33). However, such a strict inclusion criterion, would have missed our patient due to negative family history.

The most comprehensive genetic study on seronegative MG was recently conducted by Krenn et al. (36). Among 50 triple-seronegative patients confirmed by live CBA, seven (14%) received a genetic diagnosis of CMS through WES, including four with *CHRNE* and three with *RAPSN* variants. Patients with CMS tended to have a younger age at disease onset, and only one patient with confirmed CMS reported a positive family history.

A heterozygous pathogenic missense mutation, c.2368G>A, in the *MUSK* gene was identified in our second patient (37). *MUSK*-related CMS is inherited in an autosomal recessive manner. Although WGS did not reveal another pathogenic variant in our patient, the possibility of a large deletion or duplication on the other allele still remains an option. On the other side, patients with deletions in *MUSK* gene tend to have more severe, early-onset phenotype (38). Limitation of our research is that segregation analysis of the variant was not performed since family members were not available for genetic testing.

Neither DM1 nor OPMD cases were identified in our dSnMG cohort, whereas one patient was found to carry a DM2 premutation. It has been shown that patients with DM2 have a significantly higher prevalence of autoimmune disorders compared to the general population, and cases of co-occurring MG and DM2 have been previously reported (39–41). Nevertheless, our patient with DM2 premutation had a complete response to MG therapy and did not exhibit any of the symptoms attributed to DM2 at age 42. However, given that the onset of DM2 may start in the fifth decade of life (42), and that there are reported cases of symptomatic carriers of the *CNBP* gene premutation, such symptoms could still appear over time and require careful future monitoring (43).

A key limitation of our investigation is that live CBA were not performed to exclude the presence of serum antibodies against clustered AChRs, MuSK, and LRP4. In addition, comprehensive testing for VGCC antibodies by radioimmunoassay was not feasible. Although the applied WES pipeline included coverage of the mitochondrial genome, only single-nucleotide variants, insertions, and deletions with heteroplasmy levels >10% were reported. Further analysis of mitochondrial DNA in muscle tissue would likely provide additional valuable insights. Another key limitation is the retrospective collection of clinical data.

In conclusion, 9% of patients initially diagnosed with dSnMG were ultimately found to have alternative diagnoses, including LRP4 MG, LEMS, and slow-channel CMS. Misdiagnosis in dSnMG remains a significant concern, largely driven by substantial clinical, electrophysiological, and therapeutic overlap with other neuromuscular disorders. Our findings underscore the importance of systematically considering alternative diagnoses in patients with dSnMG, not only to avoid unnecessary pharmacological overtreatment, but also because disease-specific therapies are available for CMS that may meaningfully alter disease course. Prospective studies incorporating standardized clinical and electrophysiological assessments (RNS and SFEMG), alongside systematic collection of symptomatic and immunomodulatory treatment data with clearly defined response outcomes, are needed to corroborate and further expand upon the findings of this study.

## Data Availability

The raw data supporting the conclusions of this article will be made available by the authors, without undue reservation.
